# PCV2 Infection Represses the Differentiation of Light Zone Germinal Center B Cells by Inhibiting Their Interaction with Helper Cells

**DOI:** 10.3390/microorganisms13092184

**Published:** 2025-09-18

**Authors:** Tengfei Shi, Qian Du, Jiasai Kang, Haoshu Zhang, Xinru Xu, Yang Wang, Dewen Tong, Yong Huang

**Affiliations:** 1 College of Veterinary Medicine, Northwest A&F University, Yangling 712100, China; 2 Engineering Research Center of Efficient New Vaccines for Animals, Ministry of Education, Yangling 712100, China; 3 Key Laboratory of Ruminant Disease Prevention and Control (West), Ministry of Agriculture and Rural Affairs, Yangling 712100, China; 4 Engineering Research Center of Efficient New Vaccines for Animals, Universities of Shaanxi Province, Yangling 712100, China

**Keywords:** porcine circovirus type 2, B cell, differentiation, germinal center reaction, cell communication

## Abstract

Porcine circovirus 2 (PCV2) is one of the most widespread immunosuppressive viruses, impairing the protective efficacy of vaccines in pig herds. Previous studies have shown that PCV2 infection reduces the generation of immune memory and antibody secretion induced by vaccination in hosts. In this study, we used single-cell mRNA sequencing of mice splenic cells to show that PCV2 infection decelerates the differentiation of light zone germinal center (GC) B cells into memory B cells and plasma cells. We found that, although PCV2 infection led to lymphocyte depletion in the spleens of mice, the remaining splenic B cells were activated by the infection. The percentage of naïve B cells in PCV2-infected mice decreased mainly due to differentiation rather than death. Meanwhile, the percentages of memory B cells and plasma cells increased without significant enhancement of functional gene expression. Focusing on the GC B cells, we found that PCV2 infection activated the proliferation of dark zone GC B cells, but not the differentiation of light zone GC B cells. Furthermore, the transcriptional level of *Prdm1* was not significantly altered by PCV2 infection, and the level of *Bach2* was dramatically reduced. Further analysis showed that the interactions between light zone GC B cells and dendritic cells, macrophages, and follicular helper T cells were weakened in the spleens of PCV2-infected mice. In conclusion, this study found that PCV2 infection impairs the differentiation of B cells into functional memory B cells and plasma cells. This may be an important and previously unrecognized reason why PCV2 infection impairs vaccine efficiency.

## 1. Introduction

PCV2 is one of the most widespread viruses in the global pig population and has caused significant economic losses [1]. PCV2 infection often leads to immunosuppression, weakening the immune response to vaccines and reducing levels of antibody production and the duration of vaccine protection [2]. The reduction in lymphocytes in porcine lymphoid tissues due to PCV2 infection has long been recognized as the main reason for the immune system’s inability to respond to vaccines [3]. However, we have observed that some lymphocytes remain, and the ability of these remaining lymphocytes to respond largely determines the performance of host immunity [4]. B cells are the main type of lymphocytes that directly affect the humoral immunity by producing antibodies against vaccines or pathogens. However, the effect of PCV2 infection on the function and response of the remaining B cells in the host is unknown.

Following antigen stimulation, naïve B cells in the lymph nodes of peripheral tissues become activated and migrate into lymphoid follicles, where they form germinal centers (GCs) through massive proliferation [5]. The formed GCs are divided into two zones: the light zone (LZ) and the dark zone (DZ). In the DZ, B cells undergo rapid proliferation accompanied by somatic hypermutation (SHM). The proliferated cells then gradually migrate to the LZ. In the LZ, B cells exit the cell cycle and undergo affinity selection, potentially differentiating into plasma cells or memory B cells [6]. Antigen-presenting cells (APCs) and follicular helper T cells (Tfh cells) participate in the affinity maturation process of GC B cells by secreting cytokines and through direct contact [7,8]. Antigens carried by APCs, such as dendritic cells (DCs) and macrophages, can activate downstream signaling pathways directly, thereby stimulating the expression of BCL6 and IRF4 [9]. BCL6 directly or indirectly promotes the expression of activation-induced cytidine deaminase (AID), thereby promoting SHM in GC B cells. Meanwhile, IRF4 directly regulates the expression of PRDM1, causing GC B cells to differentiate into plasma cells [10]. Additionally, BCR stimulation regulates the expression of BACH2 and BCL2; high levels of these proteins induce the differentiation of GC B cells into memory B cells [11,12]. LZ GC B cells process captured antigens into antigen peptides and present them to Tfh cells. During this process, Tfh cells bind to CD40 on the surface of GC B cells via CD40L, providing a costimulatory signal and promoting the positive selection of LZ GC B cells [12,13]. Furthermore, IL21, secreted by Tfh cells, promotes the differentiation of LZ GC B cells into memory B cells or plasma cells [14]. It is still unknown whether PCV2 infection affects the corresponding B cell subsets at different stages of differentiation during B cell activation.

Here, we performed single-cell RNA sequencing (scRNA-seq) and flow cytometry analysis of spleen cells from PCV2-infected and mock-infected mice, in order to elucidate the effect of PCV2 infection on B cell development and differentiation. The results showed that PCV2 infection reduced the number of B cells. Although PCV2 infection induced the activation and differentiation of naïve B cells, like other pathogens, and the percentage of memory B cells and plasma cells produced by PCV2 stimulation was increased, the expression of their functional genes remained at the same level as the corresponding cells from mock-infected mice. This indicates that the function of B cells in PCV2-infected mice is suppressed. Further analysis revealed that PCV2 infection inhibited the interactions between LZ GC B cells and Tfh cells, DCs, and macrophages, thereby suppressing the expression of key genes that regulate LZ GC B cell differentiation. In summary, our results demonstrate the impact of PCV2 infection on B cell subsets, particularly plasma cells and memory B cells, and lay the groundwork for further research into the mechanisms underlying PCV2-induced humoral immune suppression.

## 2. Materials and Methods

Virus, cells, and animals: The PCV2 strain (GenBank accession no.MH492006), isolated and stocked in our lab, was propagated in PK-15 cells (The American Type Culture Collection, Manassas, VA, USA). The PK-15 cells were maintained in Dulbecco’s Modified Eagle Medium, supplemented with 10% heat-inactivated fetal bovine serum (Cat# FSP500, ExCell, Suzhou, China), at 37 °C with 5% CO_2_. The BALB/c mice were purchased from the Northwest A&F University Experimental Animal Center.

Mouse experiment: Ten 4-week-old BALB/c mice were randomly divided into two groups. The mice in the PCV2 group were injected intraperitoneally with 500 μL of a 10^5^ TCID_50_ PCV2 suspension to construct a PCV2-infected mouse model. The mice in the control group were injected with 500 μL of phosphate buffer saline in the same manner. On days 7, 14, and 21 after infection, blood samples were collected from the tail vein of the mice and serum was isolated for PCV2 load detection. On the 28th day after infection, the mice were euthanized and their blood, hearts, livers, spleens, lungs, and kidneys were harvested for virus load detection and histological observation.

Then, for the additional experiments, we reconstructed PCV2-infected mouse models (with three mice in PCV2 group and three mice in control group) four times using the same method as for the PCV2-infected mouse model for scRNA-seq.

(1) To determine the impact of PCV2 infection on the proportion of B cells in mice, we set up a PCV2 group and a control group of mice, with 3 mice in each group. B cells in the spleens of the mice were detected using flow cytometry. Three detection data points were obtained from three different mice in each group.

(2) To investigate the impact of PCV2 infection on B-cell activation and differentiation, we set up a PCV2 group and a control group of mice, with 3 mice in each group. The spleen lymphocytes of each mouse were divided into two parts; one part was used for detecting B cell subsets by flow cytometry, and the other part was used for sorting naïve B cells by flow cytometry. The total RNA of the sorted naive B cells was extracted and reverse-transcribed into cDNA, and the transcription levels of the selected genes were detected using quantitative PCR. Three data points were obtained from three different mice in each group.

(3) To investigate the effect of PCV2 infection on humoral immune response, we set up a PCV2 group and a control group of mice, with 3 mice in each group. The spleen lymphocytes of each mouse were divided into four parts. Three of these parts were used for flow cytometry assays of Ki-67 expression levels in GCB cells, ZFP318 expression levels in memory B cells, and IgD/IgM/IgG expression levels in plasma cells. The last part was used for flow cytometric sorting of GC B cells, and the protein of these sorted GC B cells was extracted for detecting the expression of AICDA and MHC II. The three detection data points in each group are from three different mice in each group.

(4) To investigate the effect of PCV2 infection on germinal center B cell subsets, we set up a PCV2 group and a control group of mice, with 3 mice in each group. The spleen lymphocytes of each mouse were divided into two parts, one part was used for detecting B cell subsets by flow cytometry, and the other part was used for sorting LZ GC B cells by flow cytometry. The protein of these sorted LZ GC B cells was extracted for detecting the expression of MYC and BACH2. The three detection data points in each group are from three different mice in each group.

Fluorescent quantitative PCR for PCV2 detection: To quantitatively detect PCV2, PCV2 DNA was extracted from serum and tissues using a Viral RNA/DNA Extraction Kit Ver.5.0 (Cat# 9766, Takara, Beijing, China), following the manufacturer’s protocol. The real-time PCR mixture contained 10 μL of 2 × Universal SYBR Green Fast qPCR Mix (Cat# RK21203, ABclonal, Wuhan, China), 2 μL of template, 0.5 μL of forward primer (sequence: TTGAATGTGGAGCTCCTAGAT; concentration of 10 μM), 0.5 μL of reverse primer (sequence: GCAAGGTACTCACAGCAGTAGACA; concentration of 10 μM), and 7 μL of ddH_2_O. The reaction procedure was as follows: Pre denaturation at 95 °C for 3 min, and the cyclic reaction including 95 °C denaturation for 5 s and 60 °C extension for 30 s, with a total of 45 cycles. The default values of the instrument were used for the dissolution curve parameters. Once the reaction has finished, a standard curve is drawn based on the Cq (quantification cycle) values of each sample reaction well, and the copy number of PCV2 in the test samples is calculated.

Preparation of mouse spleen tissue slices: The tissue was fixed in 4% paraformaldehyde (PFA) at room temperature for 48 h, and then embedded in paraffin. The embedded tissue was sectioned into 4 μm slices and stained with hematoxylin and eosin. The sections were then observed using an optical microscope.

Tissue dissociation and preparation of single-cell suspensions: On the 28th day post infection, one mouse was randomly selected from the control and PCV2 groups. Their spleens were prepared for single-cell suspension. A sterile, RNase-free culture dish containing an appropriate amount of calcium- and magnesium-free PBS was placed on ice. The spleen tissue was transferred into the culture dish and cut it into 0.5 mm^2^ pieces. The tissues were then washed with PBS and the non-purpose tissues, such as blood stains and fatty layers, were removed. The tissues were then dissociated into single cells in a dissociation solution (0.35% collagenase IV, 2 mg/mL papain, 120 Units/mL DNase I) in a water bath at 37 °C with shaking for 20 min at 100 rpm. Digestion was terminated by adding 1× PBS containing 10% fetal bovine serum (FBS, V/V) and pipetting five times. The resulting cell suspension was filtered by passing it through a 70 μm stacked cell strainer, then centrifuged at 300× *g* for 5 min at 4 °C. The cell pellet was resuspended in 100 μL of PBS (0.04% BSA), to which 1 mL of 1× red blood cell lysis buffer (Cat# 130-094-183, Miltenyi Biotec, Bergisch Gladbach, Germany) was added. The mixture was then incubated on ice for 10 min to lyse the remaining red blood cells. After incubation, the suspension was centrifuged at 300× *g* for 5 min at room temperature. The suspension was then resuspended in 100 μL of Dead Cell Removal MicroBeads (Cat# 130-090-101, Miltenyi Biotec), after which dead cells were removed. The suspension was then resuspended in PBS (0.04% BSA) and centrifuged at 300× *g* for 3 min at 4 °C (repeated twice). The cell pellet was resuspended in 50 μL of PBS (0.04% BSA). The overall cell viability of the control group and PCV2 group were 93.4% and 94.4%, respectively, confirmed by trypan blue exclusion. The single cell suspensions in the control and PCV2 groups were counted using a Countess II Automated Cell Counter and the concentration was adjusted to 1060 and 1070 cells/μL, respectively.

Chromium 10x Genomics library and sequencing: According to the manufacturer’s instructions of 10x Genomics Chromium Single-Cell 3′ kit (V3) (10x Genomics, Pleasanton, CA, USA), single-cell suspensions of control group and PCV2 group were loaded to 10x Chromium capturing 9665 and 6477 cells, respectively. The following cDNA amplification and library construction steps were performed. Libraries were sequenced on an Illumina NovaSeq 6000 sequencing system (Illumina, San Diego, CA, USA) (paired-end multiplexing run, 150 bp) at a minimum depth of 20,000 reads per cell.

Bioinformatics analysis: The sequencing results were demultiplexed and converted to FASTQ format using Illumina bcl2fastq software (version 2.20). Sample demultiplexing, barcode processing, and single-cell 3′gene counting was performed using the Cell Ranger pipeline (https://support.10xgenomics.com/single-cell-geneexpression/software/pipelines/latest/what-is-cell-ranger, version 3.1.0, accessed on 24 June 2024) and the scRNA-seq data were aligned to the Ensembl genome GRCm38 reference genome. A total of 9665 cells in the control group and 6477 cells in the PCV2 group were captured, respectively, and then processed using the 10x Genomics Chromium Single Cell 3′Solution. The Cell Ranger output was loaded into Seurat (version 5.1.0) for dimensional reduction [15], clustering, and analysis of the scRNA-seq data. Overall, 13,901 cells (8748 for control group and 5153 for PCV2 group) passed the quality control threshold: all genes expressed in fewer than one cell were removed; the number of genes expressed per cell was set to a low cut-off of >200 and a high cut-off of <5000; the percentage of mitochondrial-DNA-derived gene expression was set to a low cut-off of <15%; and double cells and red blood cells were removed. To visualize the data, we reduced the dimensionality of all 13,901 cells using Seurat and projected the cells into 2D space using t-SNE. The steps include the following: 1. Using the LogNormalize method of the “Normalization” function of the Seurat software (Version 5.3.0) to calculated the expression value of genes; 2. PCA (Principal component analysis) analysis was performed using the normalized expression value. Within all the PCs, the top 10 PCs were used to do clustering and t-SNE analysis; 3. To find clusters, weighted Shared Nearest Neighbor (SNN) graph-based clustering method was used. Marker genes for each cluster were identified with the Wilcoxon rank-sum test with default parameters via the FindAllMarkers function in Seurat. This selects marker genes which are expressed in more than 10% of the cells in a cluster and average log_2_(Fold Change) of greater than 0.26.

Pathway enrichment analysis: Firstly, use the FindMarkers (Version 5.3.0) function to search for differentially expressed genes in a certain cell between the control group and the PCV2 group, and define genes with an absolute value of log_2_FC greater than or equal to 2 as differentially expressed genes. Then, annotate the sequenced whole genome using org.Mm.eg.db, and then perform GO BP enrichment and KEGG enrichment using the enrichGO and enrichKEGG functions, respectively.

Gene expression analysis: to analyze the expression of genes in individual cell populations, we used Dotplot function to show the expression of different marker genes.

Differential analysis of gene expression: The differences in gene expression in some cell types between the control group and the PCV2 group were analyzed with wilcox.test. Then we used the ggplot function to draw the violin plots.

Cellchat analysis: Create the Seurat object as a CellChat object and then import it into the ligand receptor database CellChatDB.mouse. Cell communication analysis is based on Secreted Signaling, ECM-Receptor, and Cell–Cell Contact. Infer biological cell–cell communication by assigning a probability value to each interaction and conducting permutation tests.

Flow cytometry detection and isolation of cells: Spleen lymphocytes were separated using a mouse spleen lymphocyte isolation kit (Cat# P8860, Solarbio, Beijing, China). The obtained lymphocytes were incubated with the antibody in the Stain buffer (Cat# 420201, Biolegend, Santiago, CA, USA) at room temperature and in the dark for 30 min, after which they were washed with PBS and tested. The types of cells detected/sorted included GC B cells (B220+CD138-CD38-GL7+), memory B cells (B220+CD138-CD38+), plasma cells (B220+CD138+CD38-), LZ GC B cells (B220+CD138-CD38-GL7+CD86^hi^CXCR4^lo^), and DZ GC B cells (B220+CD138-CD38-GL7+CD86^lo^CXCR4^hi^). The antibodies used included PE/Cyanine7 anti-B220 (Cat# 103222, Biolegend), PE anti-CD138 (Cat# 142503, Biolegend), PerCP/Cyanine5.5 anti-CD38 (Cat# 102721, Biolegend), APC anti-GL7 (Cat# 144617, Biolegend), FITC anti-CD86 (Cat# 105109, Biolegend), APC/Cyanine7 anti-CXCR4 (Cat# 146523, Biolegend), FITC anti-Ki67 (Cat# 151211, Biolegend), APC anti-IgD (Cat# E-AB-F1189E, Elabscience, Wuhan, China), FITC anti-IgM (Cat# E-AB-F1190C, Elabscience), FITC anti-IgG (Cat# 406001, Biolegend), Anti-ZNF318 (Cat# orb1088967, Biorbyt, Cambridge, UK), and APC anti-rabbit IgG (Cat# F0111, R&D Systems, Minneapolis, MN, USA). Before incubating anti-Ki67 among the above antibodies, cells were fixed with fixative solution (Cat# CO3-02001, Bioss, Beijing, China) and permeabilized with 1× intracellular staining perm wash buffer (Cat# B205349, Biolegend) sequentially. Cells were sorted on a BD FACSAriaTM III (BD, Franklin Lakes, NJ, USA), and the other data were collected on an Agilent NovoCyte (Agilent, Beijing, China). All data were analyzed with De Novo software (Version 7-01).

Quantitative RT-PCR: Total RNA of sorted naïve B cells was extracted, and to remove contamination of genomic DNA, RNA samples were treated with RNAse-free DNAse (Cat# 4716728001, Roche, Basel, Switzerland) according to the instructions. Then the RNA samples were reverse-transcribed into cDNA by HiScript^®^ RT SuperMix for qPCR (Cat# R323-01, Vazyme, Nanjing, China). Constructed cDNA libraries were then used as templates and the SYBR Green Fast qPCR Mix (Cat# RK21203, ABclonal) was used to set up qRT-PCR reactions for the detection of some gene expression. The gene expression levels were normalized to generic house-keeping gene *Gapdh*. Primers for qRT-PCR are listed in Table S2.

Western blotting: On the 28th day post infection, GC B cells and LZ GC B cells were sorted from the mouse spleen lymphocytes in the control and PCV2 groups by flow cytometry. The cells were then lysed using RIPA buffer containing 1 mM PMSF (Cat# R0020, Solarbio), after which Protein Loading Buffer (Cat# PP117-01, Beyotime, Shanghai, China) was added to the whole-cell lysates. The mixture was then placed in a boiling water bath for 10 min. After electrophoresis on SDS gels, the whole-cell lysate was transferred from the gel to a nitrocellulose membrane. The membrane was blocked with 5% BSA in PBS for 2 h, after which the samples were incubated with the targeted antibodies overnight at 4 °C. Targets were revealed by staining with HRP secondary antibodies for 2 h at room temperature. Finally, the chemiluminescent blots were imaged using X-ray film. The antibodies used in this process included anti-MHC Class II (Cat# ab139365, abcam, Cambridge, UK), anti-MYC (Cat# ab195207, abcam), anti-AICDA (Cat# YT5566, Immunoway, Plano, TX, USA), anti-BACH2 (Cat# YN16971, Immunoway), HRP anti-Rat IgG H&L (Cat# ab205720, abcam), and HRP anti-Rabbit IgG H&L (Cat# 31460, Thermo Fisher, Waltham, MA, USA).

## 3. Results

### 3.1. PCV2 Replicates in the Mouse Spleens and Induces Severe Splenic Lesions

**Figure 1:**
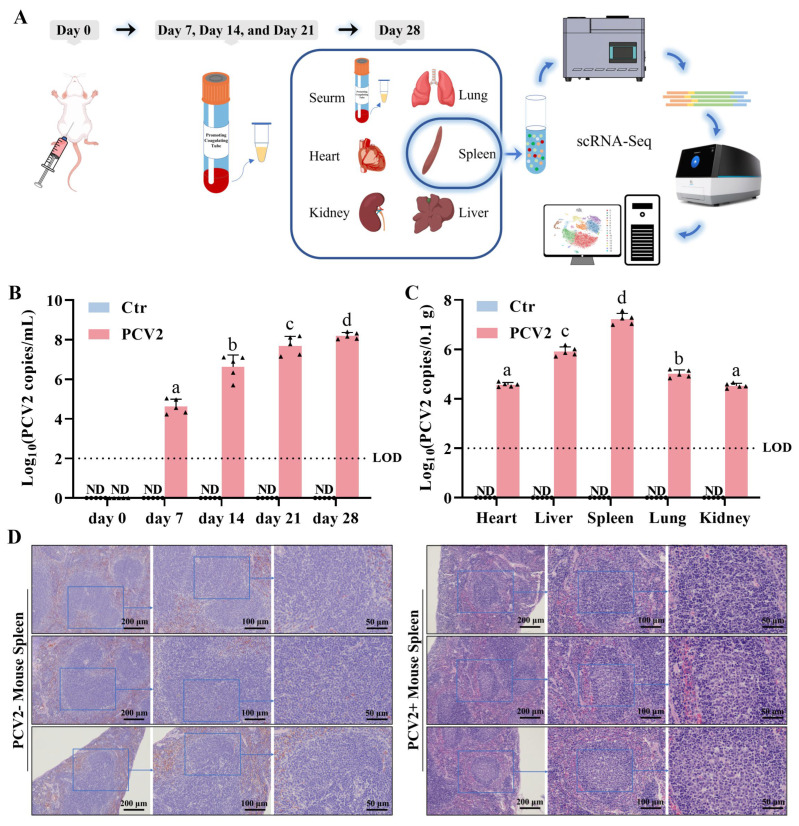
PCV2 infection mainly lesions the spleen of mice. (**A**) Schematic of the experimental design. A PCV2-infected mouse model was established by intraperitoneally injecting 500 μL of a 10^5^ TCID_50_ PCV2 suspension. Mice in the control group were simulated to undergo the same procedure with 500 μL of PBS buffer. On days 7, 14, and 21 post infection, blood samples were collected from the tail vein of the mice, serum was isolated, and the PCV2 load was detected. On the 28th day post infection, the mice were euthanized and their blood, hearts, livers, spleens, lungs, and kidneys were harvested to detect the viral load in the blood and organs. Pathological tissue observation and scRNA-seq were performed on the mouse spleens. (**B**) The PCV2 load in peripheral blood of the mice of the control and PCV2 groups during the experiment. (**C**) The PCV2 load in the organs of the mice of the control and PCV2 groups on the 28th day post infection. (**D**) Observation of mouse spleen tissue slices on the 28th day after infection. Different letters above bars denote significant differences (*p* < 0.05); shared letters indicate no significant difference. ND means not detected.

It has been reported that PCV2 primarily targets immune cells in the host and that infection alters the function of these cells. To clarify how PCV2 infection affects the antibody production in the host, we constructed a PCV2-infected mouse model and performed scRNA-seq on the spleens (Figure 1A). On the 7th day post infection, the body weight of PCV2-infected mice was significantly lower than that of the control group (Figure S1). In addition, four out of five mice on the 7th day post infection and two out of five mice on the 14th day post infection had a rectal temperature exceeding 38.5 °C (Table S1). All mice in the PCV2 group showed PCV2 viremia on the 7th day post infection, with increasing serum PCV2 loads during the experimental period reaching approximately 10^8^ copies/mL by the 28th day (Figure 1B). PCV2 loads in the spleen were the highest among the other detected organs of the mice on the 28th day post infection (Figure 1C). The pathological changes in the spleens of the mice were then checked, and the results showed that the lymphocytes were more sparse near the germinal center of the spleens of the PCV2-infected mice than of the control mice (Figure 1D). In summary, we generated a mouse model to study the lymphocytic response to PCV2 infection, and confirm that PCV2 mainly replicates in the spleen of the mouse, resulting severe splenic lesions.

### 3.2. Single-Cell RNA Sequencing Reveals That PCV2 Infection Leads to the Reduction in Lymphocytes but Also Activates the Response to Virus of B Cells in Mouse Spleen

**Figure 2:**
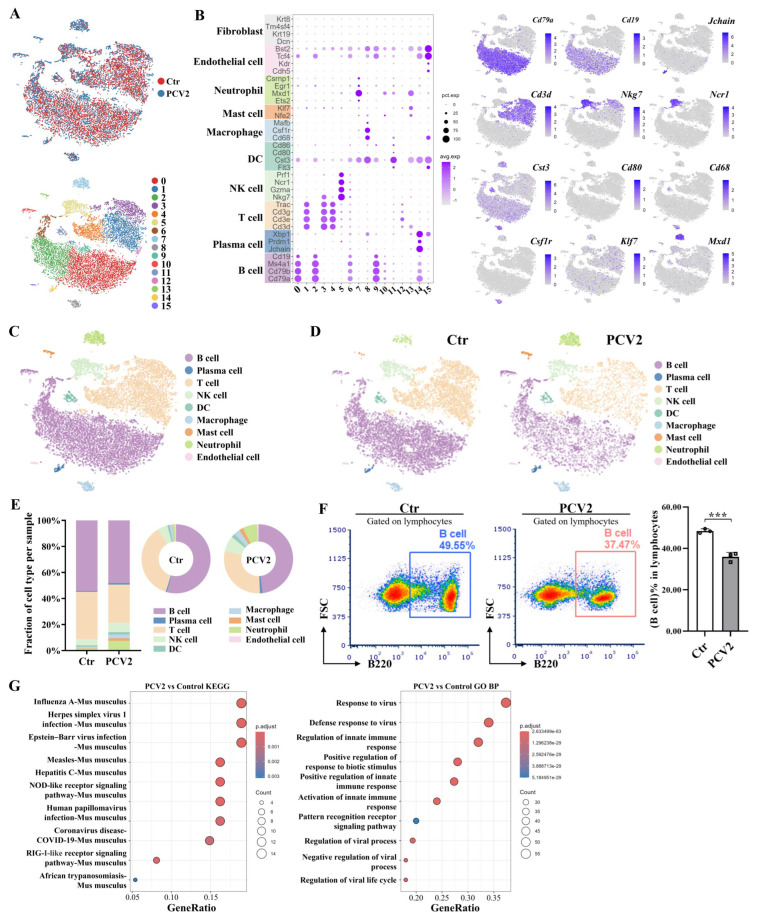
PCV2 infection reduces splenic lymphocytes, but also activates the response to the virus in the remaining B cells. (**A**) t-distributed stochastic neighbor embedding (t-SNE) plot showing the sample integration effect (above; each point represents a cell, with red representing the control group and blue representing the PCV2 group) and clustering of cells (below; different colors represent different clusters) in mouse spleen. (**B**) The expression of marker genes for multiple cell types in different clusters. The marker genes for different cell types on the left side of the bubble chart are marked with different colors as backgrounds, and the corresponding cell types are labeled next to them. t-SNE projection of some marker genes, including *Cd79a* and *Cd19* for B cells; *Jchain* for plasma cells; *Cd3d* for T cells; *Nkg7* and *Ncr1* for NK cells; *Cst3* and *Cd80* for DCs; *Cd68* and *Csf1r* for macrophages; *Klf7* for mast cells; and *Mxd1* for neutrophils as indicated in the legend. (**C**) A t-SNE plot showing B cells, plasma cells, T cells, NK cells, DCs, macrophages, mast cells, neutrophils, and endothelial cells identified using integrated and classification analysis. (**D**) The t-SNE plot shows a comparison of the cell type distribution in the control and PCV2 groups. (**E**) A comparison of the relative frequencies of the cell types in the spleen of the control and PCV2 groups. (**F**) Flow cytometry analysis of the proportion of B cells (B220+) in the mouse spleen lymphocytes of the control and PCV2 groups on the 28th day after infection. The gate of the control group is in blue, and the gate of the PCV2 group is in red. (**G**) Kyoto Encyclopedia of Genes and Genomes (KEGG) and Gene Ontology Biological Process (GO BP) enrichment analysis of the differentially expressed genes (DEGs) of B cells between the control and PCV2 groups. *** *p* < 0.001.

In order to investigate the effect of PCV2 infection on the immune response in the mouse spleens, we analyzed the scRNA-seq data from control and PCV2 group mice. Firstly, we performed strict, high-quality filtering of the sequencing data. After removing double cells and the batch effect, the scRNA-seq data from two groups could be merged well (Figure 2A). Using t-distributed stochastic neighbor embedding (t-SNE), the mouse splenic cells were divided into 16 clusters (Figure 2A). Then, we analyzed the expression of marker genes for mouse B cells, plasma cells, T cells, natural killer (NK) cells, DCs, macrophages, mast cells, neutrophils, endothelial cells, and fibroblasts in the 16 clusters (Figure 2B), and annotated these cells in the mouse spleens (Figure 2C). To investigate the effect of PCV2 infection on the composition of mouse splenic cells, we analyzed the proportion of the annotated cell populations in the spleens of the control and PCV2 group mice. The results showed that, compared to the control group cells, the percentages of B cells and T cells in the PCV2 group cells decreased, while the proportion of other immune cells increased (Figure 2D,E). Flow cytometry results further confirmed that PCV2 infection led to a significant decrease in the proportion of B cells in lymphocytes (Figure 2F). As B cells appear to be the cells most affected by PCV2 infection, we compared the mRNA expression in B cells from the control and PCV2 groups. The results showed that the differentially expressed mRNAs in B cells were mainly enriched in the viral-infection-related signaling pathways, NOD-like receptor signaling pathway, RIG-like receptor signaling pathway, antiviral response-related signaling pathways, innate immune response, and signaling pathways associated with regulation of viral biological processes (Figure 2G). Based on these results, we conclude that PCV2 infection primarily induces a reduction in lymphocytes, especially B cells in the mouse spleen, and the infection activates the responses to viruses in B cells.

### 3.3. PCV2 Infection Activates the Differentiation of Naïve B Cells and Shifts the Proportion Balance of the B Cell Subsets

**Figure 3:**
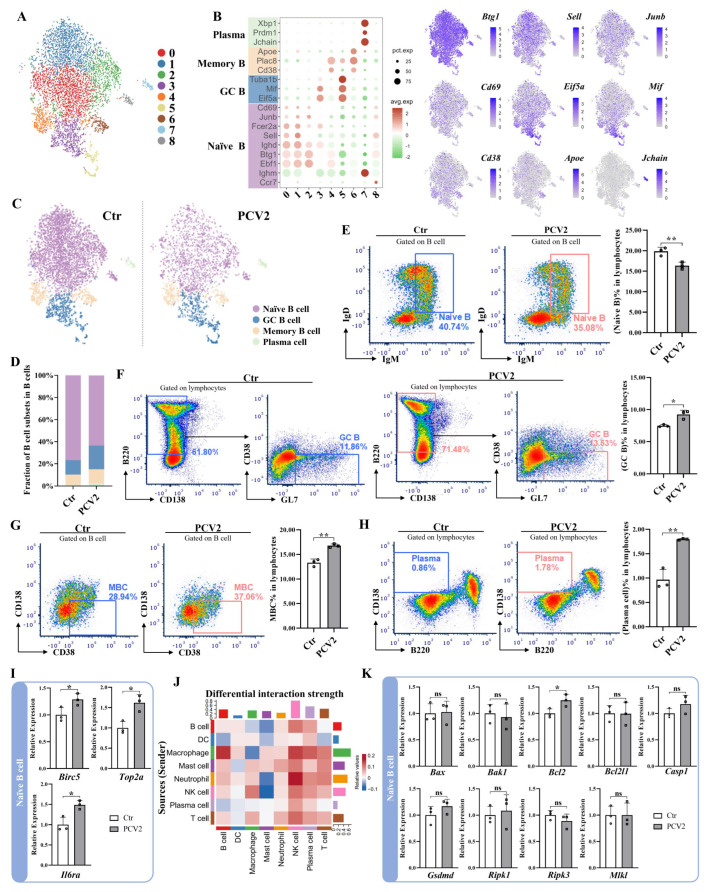
PCV2 infection leads to the differentiation but not cell death of the mouse splenic naïve B cells. (**A**) A t-SNE plot shows the clustering of B cells in the mouse spleen. (**B**) The expression of marker genes for B cell subsets in different clusters. The different B cell subsets are marked with different colors in the bubble chart on the left, and the corresponding subset is labeled next to it. t-SNE projection of some marker genes, including *Btg1*, *Sell*, *Junb*, and *Cd69* for naïve B cells; *Eif5a* and *Mif* for GC B cells; *Cd38* and *Apoe* for memory B cells; and *Jchain* for plasma cells as indicated in the legend. (**C**) A t-SNE plot showing naïve B cells, GC B cells, memory B cells, and plasma cells between the control and PCV2 groups, as identified by integrated and classification analysis. (**D**) Comparison of the relative frequencies of the B cell subsets in the spleen B cells of the control and PCV2 groups. Flow cytometry comparison of the fraction of naïve B cells (**E**), GC B cells (**F**), memory B cells (**G**), and plasma cells (**H**) in spleen lymphocytes between the control group and the PCV2 group on the 28th day after infection. (**I**) mRNA expression of *BIRC5*, *Top2a*, and *Il6ra* in spleen naive B cells sorted by flow cytometry between the control and PCV2 groups on the 28th day after infection. The average level in the control group is set to 1. Three samples were tested in each group. (**J**) Cell interaction intensity heatmap of the PCV2 group. The vertical axis represents cells that emit signals and the horizontal axis represents cells that receive signals. The color intensity of the heatmap represents the signal strength. The columns on the upper and right sides are the cumulative strength of the vertical and horizontal axes. Red indicates upregulation of the PCV2 group relative to the control group, while blue indicates downregulation. (**K**) mRNA expression in spleen naive B cells between the control and PCV2 groups on the 28th day after infection. The average level in the control group is set to 1. Three samples were tested in each group. The gates of the control group are in blue, and the gates of the PCV2 group are in red (**D**–**H**). * *p* < 0.05, ** *p* < 0.05, and ns means not significant.

To further analyze the effect of PCV2 infection on B cell subsets, we isolated splenic B cells and plasma cells, separating them from other cells, and divided these cells into nine clusters (Figure 3A). We then analyzed the expression of marker genes for mouse B cell subsets (naïve B cells, GC B cells, memory B cells, and plasma cells). The results showed that clusters 0, 1, 2, and 8 specifically expressed mouse naïve B cell marker genes, so these clusters were annotated as naive B cells (Figure 3B,C). Similarly, clusters 3 and 5 were annotated as GC B cells; cluster 4 and 6 as memory B cells; and cluster 7 as plasma cells (Figure 3B,C). When cells from the control and PCV2 groups were separated, it was clearly shown that the proportion of naïve B cells in total B cells decreased in the PCV2-infected mice compared to control mice, while the proportions of GC B cells, memory B cells, and plasma cells were increased (Figure 3C,D). Flow cytometry results further confirmed that PCV2 infection significantly reduced the proportion of naïve B cells in spleen lymphocytes (Figure 3E) and increased the proportions of GC B cells (Figure 3F), memory B cells (Figure 3G), and plasma cells (Figure 3H) in spleen lymphocytes. To determine the possible reason for the reduction in the proportion of naïve B cells induced by PCV2 infection, we analyzed and tested the expression levels of some differentiation-related genes (*Birc5* and *Top2a*) and a cytokine receptor gene (*Il6ra*). We found that PCV2 infection significantly upregulated the expression of all these genes (Figure S2A and Figure 3I). Meanwhile, PCV2 infection enhanced the interaction intensity of B cells with other immune cells in the mouse spleen (Figure 3J). To clarify whether the decrease in the total number of B cells is related to the death of naïve B cells, we analyzed and tested the expression levels of genes relating to different programed cell death (the apoptosis-positive regulation-associated genes *Bax* and *Bak1*, the apoptosis-negative regulation-associated genes *Bcl2* and *Bcl2l1*, the pyroptosis-positive regulation-associated genes *Casp1* and *Gsdmd*, the necroptosis-positive regulation-associated genes *Ripk1*, *Ripk3*, and *Mlkl*) in the naïve B cells. The results showed that, except for *Bcl2* expression being significantly elevated in naïve B cells of PCV2-infected mice compared with control cells, the expression levels of the other genes did not change significantly (Figure S2B and Figure 3K). The differentially expressed genes in naive B cells between the control and PCV2 groups were mainly enriched in signaling pathways related to viral infection, the innate immune response, the antiviral response, and the regulation of viral life processes. No death-related signaling pathways were found among the enriched signaling pathways (Figure S2C,D). These results demonstrate that PCV2 infection induces the differentiation of naïve B cells without causing their death.

### 3.4. PCV2 Infection Promotes the Proliferation of Germinal Center B Cells but Does Not Enhance the Functional Gene Expression Levels of the Memory B Cells and Plasma Cells

**Figure 4:**
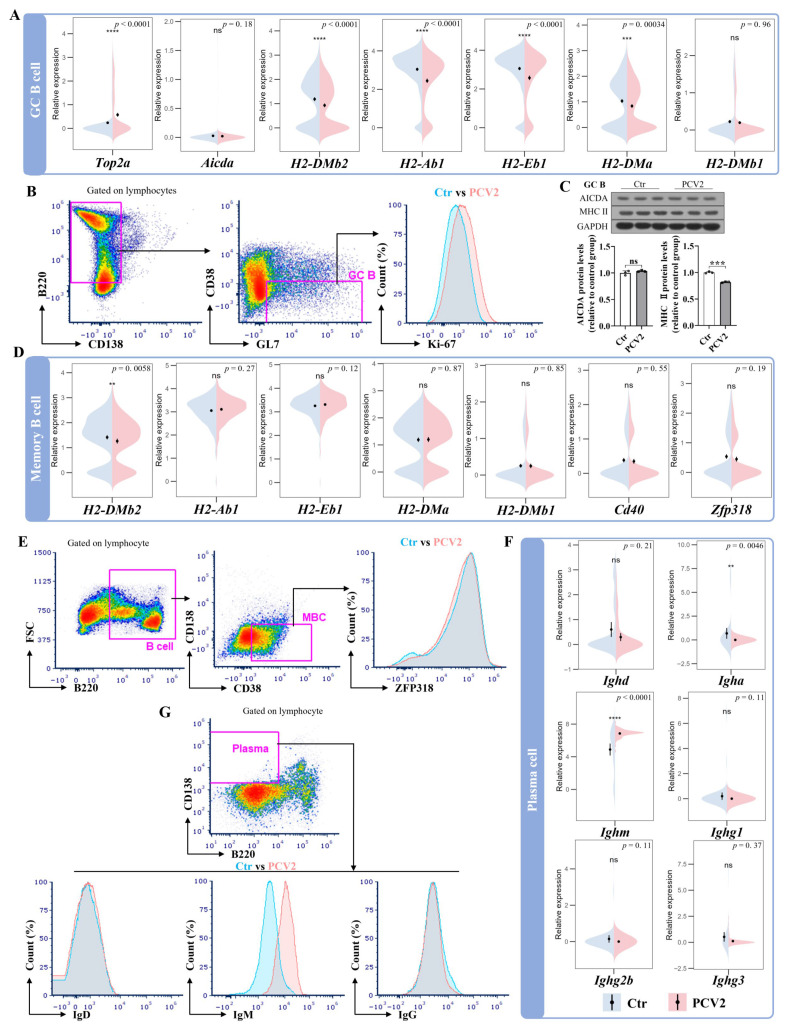
PCV2 infection promotes the proliferation of germinal center B cells but does not enhance the function of the memory B cells and plasma cells. (**A**) The violin plot shows the comparative expression levels of *Top2a* (cell replication-related gene), *Aicda* (somatic hypermutation-related gene) and *H2-DMb2*, *H2-Ab1*, *H2-Eb1*, *H2-DMa*, and *H2-DMb1* (MHC II-related genes) in GC B cells between the control and PCV2 groups. (**B**) Flow cytometry analysis of the GC B cell division ability through Ki-67 expression between the control and PCV2 groups on the 28th day after infection. (**C**) AICDA and MHC II protein expression in spleen GC B cells of the control and PCV2 groups on the 28th day after infection. (**D**) The violin plot displays the comparative results of the expression of MHC II-related genes *H2-DMb2*, *H2-Ab1*, *H2-Eb1*, *H2-DMa*, and *H2-DMb1*, the cell response ability-related gene *Cd40*, and the recall ability-related gene *Zfp318* in memory B cells between the control and PCV2 groups. (**E**) Flow cytometry analysis of the memory B cell (MBC) recall ability through ZFP318 expression between the control and PCV2 groups on the 28th day after infection. (**F**) The violin plot showing the comparative expression of immunoglobulin-related genes, *Ighd*, *Igha*, *Ighm*, *Ighg1*, *Ighg2b*, and *Ighg3*, in plasma cells between the control and PCV2 groups. (**G**) Flow cytometry analysis of the expression of IgM, IgD, and IgG in spleen plasma cells between the control and PCV2 groups on the 28th day after infection. ** *p* < 0.01, *** *p* < 0.001, **** *p* < 0.0001, and ns means not significant.

As PCV2 infection was found to activate the differentiation of naïve B cells and increase the proportions of GC B cells, memory B cells, and plasma cells, the key functional gene expression levels in these B cell subsets were explored in mice from the PCV2 and control groups. In GC B cells, the expression levels of genes related to cell proliferation (*Top2a*, *Pcna*, *Pclaf*, and *Hmgb2*) were significantly higher in the PCV2 group than in the control group (Figure 4A and Figure S3A). Higher Ki-67 expression at the protein level further indicated that PCV2 infection stimulates the division activity of GC B cells (Figure 4B). The expression level of one of the SHM-related genes, *Fam72a*, was increased by PCV2 infection (Figure S3A), but the expression level of a more important gene, *Aicda*, was not altered by PCV2 infection at the transcriptional or protein levels (Figure 4A,C). Furthermore, PCV2 infection significantly decreased the expression levels of *Ighd* and *Ighm*, but did not influence the expression of *Ighg1*, *Ighg2b*, and *Ighg3* in GC B cells (Figure S3A). The genes related to the response ability of GC B cells (*Irf4* and *Cd40*) were also not significantly altered by PCV2 infection (Figure S3A). However, the expression levels of most MHC II molecules in the transcriptional level (*H2-DMb2*, *H2-Ab1*, *H2-Eb1*, and *H2-DMa*) and protein level were reduced by PCV2 infection (Figure 4A,C). In memory B cells, PCV2 infection significantly suppressed the expression of *Ighd*, *Igha*, and *H2-DMb2*. However, the expression levels of the other immunoglobulins, MHC II molecules, as well as the immune response ability-related gene *Cd40* and the recall ability of memory B cell representative gene *Zfp318* were significantly affected by PCV2 infection (Figure S3B and Figure 4D). The unchanged ZFP318 protein expression level in memory B cells emphasizes that PCV2 does not alter the recall ability of memory B cells (Figure 4E). In plasma cells, PCV2 infection reduced the expression of *Igha*, upregulated the expression of *Ighm*, and did not significantly affect the expression of *Ighd*, *Ighg1*, *Ighg2b*, and *Ighg3* (Figure 4F). At the protein level, PCV2 infection only increased the expression level of IgM, with no alteration observed in the expression of IgD and IgG in plasma cells (Figure 4G). These results indicate that PCV2 infection induces the proliferation of GC B cells, but suppresses their antibody class switch. More importantly, the recall ability of memory B cells and the antibody production ability of plasma cells are not enhanced as with other antigen stimulations.

### 3.5. PCV2 Infection Makes LZ GC B Cells More Inclined to Differentiate into Recycling GC B Cells but Not Function Cells

**Figure 5:**
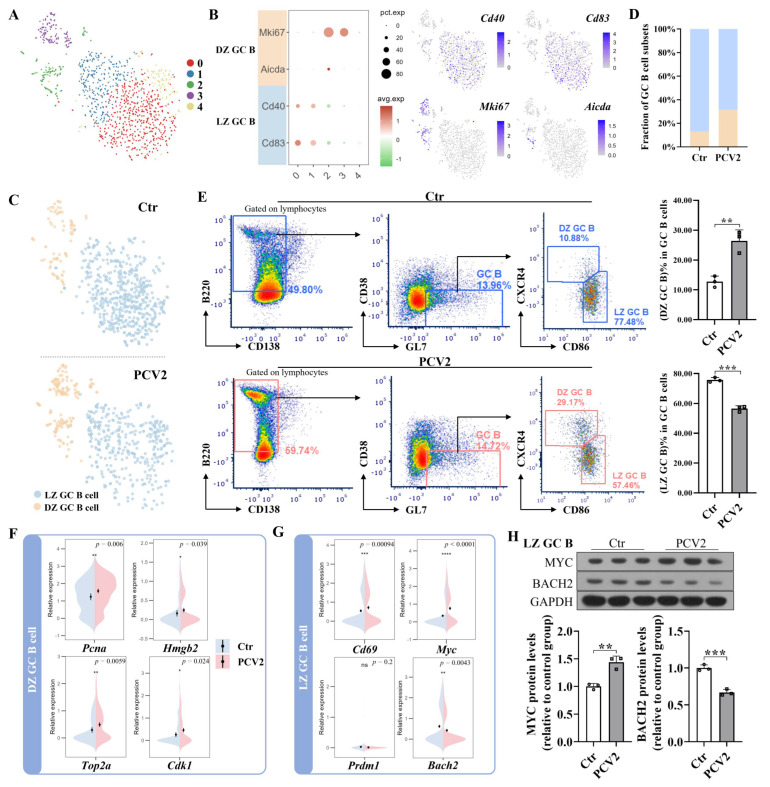
PCV2 infection promotes the proliferation of DZ GC B cells while failing to induce the LZ GC B cells to differentiate into function cells. (**A**) t-SNE plot showing the clustering of GC B cells in the mouse spleen. (**B**) The expression of marker genes for GC B cell subsets in different clusters. The marker genes for different GC B cell subsets are marked with different colored backgrounds on the left side of the bubble chart, with the corresponding subsets labeled next to them. t-SNE projection of the marker genes, including *Cd40* and *Cd83* for LZ GC B cells; and *Mki67* and *Aicda* for DZ GC B cells as indicated in the legend. (**C**) t-SNE plot showing LZ GC B cells and DZ GC B cells in the control and PCV2 groups, identified using integrated and classification analysis. (**D**) Comparison of the relative frequencies of the GC B cell subsets in the spleens of the control and PCV2 groups. (**E**) Flow cytometry analysis of the fraction of GC B cell subsets in GC B cells in the control and PCV2 groups on the 28th day after infection. The gates of the control group are in blue, and the gates of the PCV2 group are in red. (**F**) Violin plot showing the comparative expression of the characteristic gene *Pcna*, *Hmgb2*, *Top2a*, and *Cdk1* of cell replication in the DZ GC B cells between control group and PCV2 group. (**G**) Violin plot showing the comparative expression of the characteristic gene *Cd69* of B cell activation and genes regulating the differentiation of LZ GC B cells into recycling GC B cells (*Myc*), memory B cells (*Bach2*), and plasma cells (*Prdm1*) in the LZ GC B cells between the control group and the PCV2 group. (**H**) MYC and BACH2 protein expression in the LZ GC B cells of the spleens in the control and PCV2 groups on the 28th day after infection. * *p* < 0.05, ** *p* < 0.01, *** *p* < 0.001, **** *p* < 0.0001, and ns means not significant.

Germinal center response determines the performance of memory B cells and plasma cells. Thus, we isolated the GC B cells from other B cell subsets and annotated them as LZ GC B cells or DZ GC B cells based on the expression of marker genes (Figure 5A–C). Upon separating the control group cells and the PCV2 infection group cells, we found that the proportion of LZ GC B cells among the GC B cells in the PCV2 group was significantly lower than that in the control group, while the proportion of DZ GC B cells was the opposite (Figure 5C–E). To further investigate the effect of PCV2 infection on DZ GC B cells and LZ GC B cells, we analyzed the gene expression in these cells. The results showed that the expression levels of the cell replication-related genes *Pcna*, *Hmgb2*, *Top2a*, and *Cdk1* in DZ GC B cells from the PCV2 group were significantly higher than in cells from the control group (Figure 5F). Furthermore, we found that PCV2 infection significantly upregulated the expression levels of the B cell activation gene *Cd69* and the recycling GC B cell differentiation regulatory gene *Myc* in LZ GC B cells, yet did not affect the expression level of the plasma cell differentiation regulatory gene *Prdm1*, while significantly decreasing the expression level of the memory B cell differentiation regulatory gene *Bach2* (Figure 5G). We also confirmed that PCV2 infection increased MYC protein expression and decreased BACH2 protein expression levels in LZ GC B cells (Figure 5H). Based on these results, we propose that PCV2 infection activates GC B cells to proliferate in the dark zone, but does not induce them to differentiate into memory B cells and plasma cells in the light zone.

### 3.6. PCV2 Infection Weakens the Signals from Helper Cells to LZ GC B Cells and Memory B Cells

**Figure 6:**
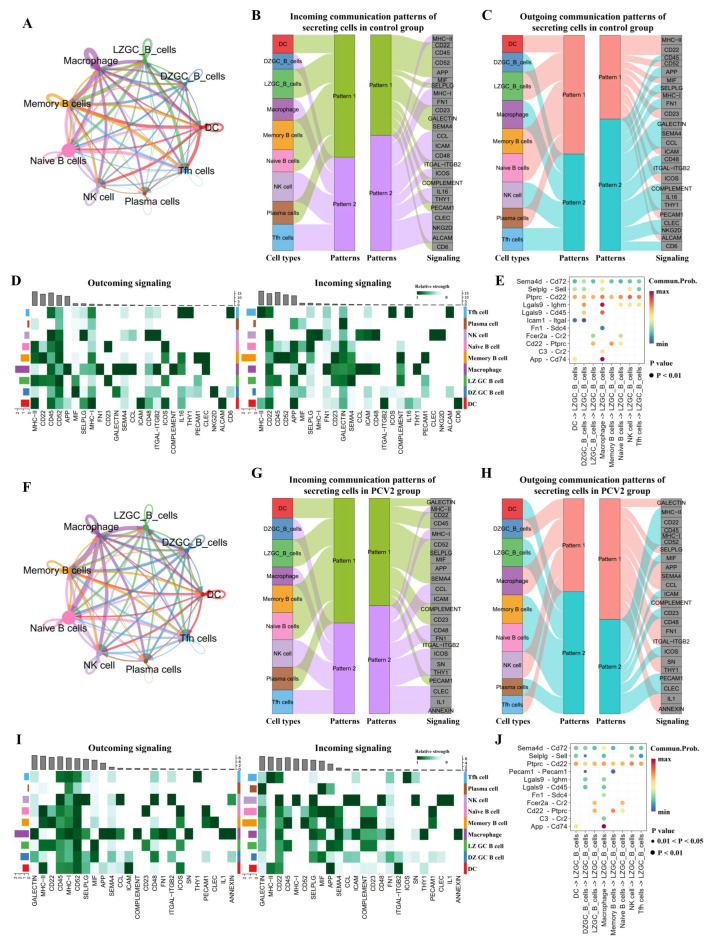
The B cell subsets of both control and PCV2 groups interact with multiple helper cells. (**A**) Cellular interaction strength in the control group. The size of the colored circles around the periphery indicates the number of cells, with larger circles indicating a great number of cells. Cells that emit arrows express ligands, while cells pointed to by arrows express receptors. The thicker the line, the higher the probability of interaction. (**B**) The inferred incoming communication patterns of secreting cells and the correspondence between the inferred latent patterns, cell groups, and signaling pathways in the control group. The thickness of the flow indicates the contribution of the cell group or signaling pathway to each latent pattern. (**C**) The inferred outgoing communication patterns of target cells in the control group. (**D**) Identifying the signal flow pattern of cells in the control group. The horizontal axis of the figure represents different pathways, and the vertical axis represents the cell types. The left figure shows the intensity of signals emitted by various pathways in different cell types, and the right figure shows the intensity of signals received by various pathways in different cell types. (**E**) Regulatory effects of signaling pathways from different cells on LZ GC B cells in the control group. (**F**) Cellular interaction strength in the PCV2 group. (**G**) The inferred incoming communication patterns of target cells in the PCV2 group. (**H**) The inferred outgoing communication patterns of target cells in the PCV2 group. (**I**) Identifying the signal flow pattern of cells in the PCV2 group. (**J**) Regulatory effects of signaling pathways from different cells on LZ GC B cells in the PCV2 group. The color of the dots represents different cell types, with corresponding cell type names marked next to them. The line of the same color as the dot represents the signal emitted from this cell population, which is the ligand signal provided by these cells (**A**,**F**).

The above results showed that, although PCV2 infection activates B cells to differentiate, it does not induce effective humoral immune responses in functionally distinct B cell subsets. Tfh cells, DCs, and macrophages are essential for B cell differentiation and function, through communicating with B cells. We analyzed and compared their intercellular interactions with different B cell subsets. LZ GC B cells in the control group received the most signals from macrophages, followed by DCs, and then Tfh cells and NK cells (Figure S4 and Figure 6A). The signals received by memory B cells in the control group mostly came from macrophages, followed by DCs, NK cells, and Tfh cells (Figure 6A). Meanwhile, memory B cells exhibited the highest signal intensity of all the analyzed B cell subsets in the control group (Figure 6A). Global communication patterns and key signals in different cell groups were analyzed and identified. The results revealed two patterns for incoming signaling (Figure 6B) and two patterns for outgoing signaling (Figure 6C) in control group cells. The communication patterns of target cells show that the incoming signaling of LZ GC B cells and memory B cells is dominated by pattern 1, which includes signaling pathways such as CD22, CD23, GALECTIN, SEMA4, ITGAL-ITGB2, COMPLEMENT, PECAM1, and CD6 among others (Figure 6B). On the other hand, this output showed that outgoing DC signaling is characterized by pattern 1, representing multiple pathways, including MHC II, CD22, MHC I, ICAM, ICOS, and PECAM1 (Figure 6C). The outgoing signaling of macrophages and Tfh cells is all characterized by pattern 2, representing pathways such as CD45, FN1, GALECTIN, SEMA4, CCL, CD48, ITGAL-ITGB2, COMPLEMENT, and CD6 (Figure 6C). The outgoing signals of LZ GC B cells in the control group mainly include PECAM1, ICOS, CD23, MIF, CD52, CD45, CD22, and MHC II, etc., and the main incoming signals are PECAM1, COMPLEMENT, SEMA4, GALECTIN, CD23, APP, CD45, and CD22, etc. (Figure 6D). The main outgoing signals from memory B cells in the control group are CLEC, PECAM1, IL16, ICOS, MHC I, CD52, CD45, CD22, and MHC II, etc., and the main incoming signals are PECAM1, COMPLEMENT, SEMA4, GALECTIN, CD23, SELPLG, APP, CD52, CD45, and CD22, etc. (Figure 6D). By systematically investigating the predicted placode-to-LZ GC B cell signals, we found 11 ligand–receptor pairs implicating SEMA, SELPLG, PTPRC, LGALS9, ICAM1, FN1, FCER2, CD22, C3, and APP signaling pathways in the process of LZ GC B cell specification (Figure 6E).

The trend in signal intensity received by B cell subsets from Tfh cells, DCs, macrophages, and NK cells in the PCV2 group was different to that in the control group. The highest signal intensity came from macrophages to LZ GC B cells in the PCV2 group, followed by Tfh cells, NK cells, and DCs (Figure 6F). Memory B cells in the PCV2 group received the strongest signal intensity from macrophages, followed by Tfh cells, NK cells, and DCs (Figure 6F). Incoming signaling to LZ GC B cells and memory B cells in the PCV2 group was dominated by pattern 1, whose signaling pathways were similar to those in the control group except for MIF (Figure 6B,G). Outgoing DC signals were characterized by pattern 2, representing multiple pathways similar to pattern 1 in the control group, except for CD45 and CLEC (Figure 6C,H). The outgoing signals of macrophages and Tfh cells were all characterized by pattern 1, similar to pattern 2 in the control group, except for SN, IL1, and ANNEXIN (Figure 6C,H). The main outgoing signals of LZ GC B cells in the PCV2 group were ICOS, CD23, MIF, CD52, MHC I, CD45, CD22, and MHC II, etc., and the main incoming signals included CD23, COMPLEMENT, SEMA4, APP, SELPLG, CD45, CD22, and GALECTIN, etc. (Figure 6J).

**Figure 7:**
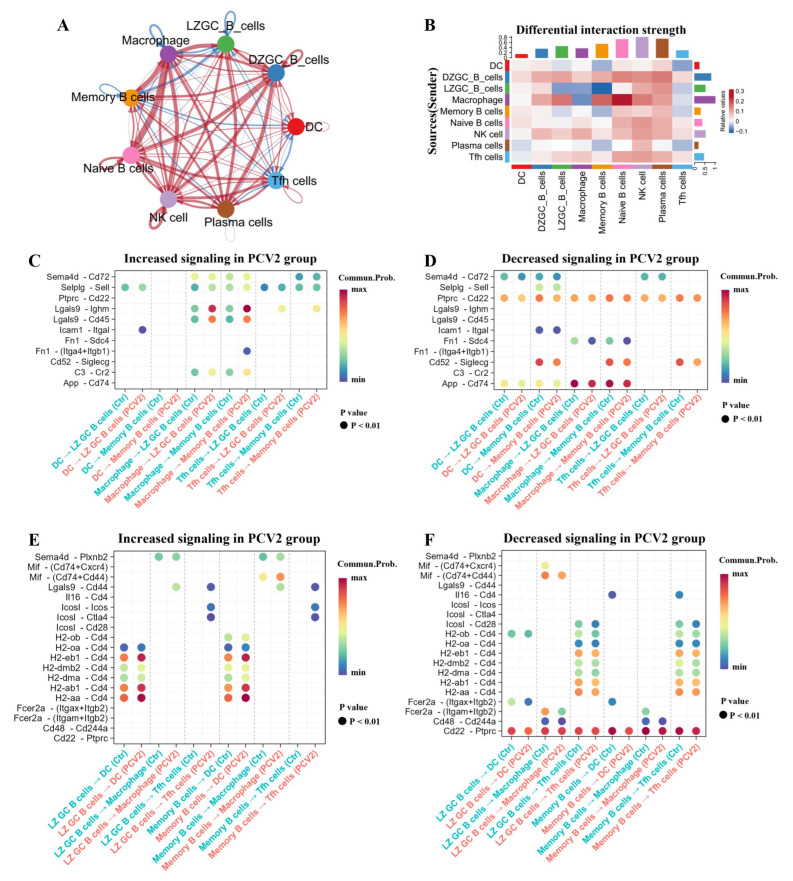
PCV2 infection weakens the interaction between B cell subsets and the helper cells. (**A**) Network diagram showing differences in cellular interaction strength between the PCV2 group and the control group. Red arrows indicate upregulation in the PCV2 group relative to the control group, while blue arrows indicate downregulation. (**B**) Heatmap showing the differences in cellular interaction strength between the PCV2 group and the control group. (**C**) Dot plot showing increased ligand–receptor interaction from DCs, macrophages, and Tfh cells to LZ GC B cells and memory B cells in the PCV2 group compared to the control group. The color of the dots reflects communication probabilities and their size represents computed *p*-values. An empty space means the communication probability is zero. *p*-values are computed from a one-sided permutation test. Only when there is a difference in ligand–receptor pairs between the control group and the PCV2 infection group are the corresponding points marked at the intersection of the *x*-axis and *y*-axis. (**D**) Dot plot showing decreased ligand–receptor interactions from DCs, macrophages, and Tfh cells to LZ GC B cells and memory B cells in the PCV2 group compared to the control group. (**E**) Dot plot showing increased ligand–receptor interaction from LZ GC B cells and memory B cells to DCs, macrophages, and Tfh cells in the PCV2 group compared to the control group. (**F**) Dot plot showing decreased ligand–receptor interaction from LZ GC B cells and memory B cells to DCs, macrophages, and Tfh cells in the PCV2 group compared to control group.

Furthermore, we compared the intensity of cell signal exchange between the PCV2 group cells and control group cells. The results showed that, compared with the control group cells, signals from DCs to LZ GC B cells, from LZ GC B cells to Tfh cells and macrophages, from DCs to memory B cells, from memory B cells to Tfh cells, and from plasma cells to DCs and Tfh cells were all weakened (Figure 7A,B). Compared to the control group cells, most signals from DCs to LZ GC B cells and memory B cells in the PCV2 group were weakened (Figure 7C,D). Among the signals from macrophages to LZ GC B cells and memory B cells, the Lgals9-Ighm/Cd45 signaling showed a greater enhancement by PCV2 infection, while those signals that were originally stronger in the control group, such as Ptprc-Cd22, Cd52-Siglecg, and App-Cd74, were weaker (Figure 7C,D). Among the signals from LZ GC B cells to DCs, multiple MHC II protein complex subunits exhibited enhanced interaction with Cd4, while signals including Fcer2a-(Itgax+Itgb2) and Cd22-Ptprc were weakened by PCV2 infection (Figure 7E,F). The signals emitted by LZ GC B cells to macrophages and Tfh cells were mostly attenuated by PCV2 infection (Figure 7E,F). The trend of signaling transmission from memory B cells to DCs, macrophages, and Tfh cells was similar to that of signaling transmission from LZ GC B cells in the PCV2 group (Figure 7E,F). In summary, PCV2 infection weakens the information exchange between DCs, macrophages, and Tfh cells with LZ GC B cells. This may be the main reason why PCV2 infection inhibits the differentiation of LZ GC B cells into functional memory B cells and plasma cells.

## 4. Discussion

Multiple studies have reported that PCV2 infection leads to a decrease in lymphocytes, which is closely related to the immune suppression caused by PCV2 [3]. Nevertheless, the performance of the remaining lymphocytes reflects the body’s immune capacity. The effects of PCV2 infection on B cells at different activation stages, as well as the performance of memory B cells and plasma cells, remain unknown. We constructed a PCV2-infected mouse model and compared histological observations of the spleens in the control and PCV2 groups. The lymphoid follicles in the spleens of control group mice were almost entirely in a primary state, and the cells were tightly arranged. In contrast, the spleens of PCV2-infected mice contained secondary lymphoid follicles and the lymphocytes in these follicles were sparse. As the spleen is a key site for B cell activation and the production of plasma cells and memory B cells [16], we performed scRNA-seq and flow cytometry on spleen cells from these mice. The results of the analysis showed that PCV2 infection reduces the number of splenic lymphocytes, especially B cells. Plasma cells and memory B cells generated by PCV2-stimulated B cell activation perform poorly due to an insufficient germinal center response. Furthermore, PCV2 infection promoted the differentiation of recycling GC B cells into LZ GC B cells while hindering the production of plasma cells and memory B cells. This may be due to the weakening of key interaction signals between LZ GC B cells and Tfh cells, DCs, and macrophages, resulting in impaired differentiation and antibody production.

The spleen is one of the main organs involved in activating B cells to produce plasma cells and mediate humoral immunity [16]. Studies have showed that B cells account for around 50% of total cells in the mouse spleen. Under normal circumstances, B cells rapidly activate and proliferate in response to antigens [5]. However, PCV2 infection activated B cells, yet led to a 6.15% decrease in their proportion within the spleen. Furthermore, PCV2 infection mobilized the entire immune system response, activating and reducing naive B cells and shifting the balance of the proportion of each B cell subgroup. The reason for this was not the death of naive B cells but the differentiation into downstream B cell subsets. PCV2 infection enhanced GC B cell proliferation but did not significantly alter SHM levels. Together with the limited antibody diversity in plasma cells, this suggests that PCV2 preferentially induces GC-independent plasma cell differentiation.

PCV2 infection reduces the expression levels of MHC II in GC B cells, thereby affecting their ability to present antigens, which is crucial for their own development. IRF4 is a key factor in B cell development [17]. At low concentrations, IRF4 promotes the upregulation of AID expression [18,19], contributing to GC B cell formation and antibody affinity maturation. At higher concentrations, IRF4 regulates the expression of the Blimp-1 TF, promoting plasma cell differentiation [20]. CD40 plays an important role in providing positive selection-related signals from Tfh cells to GC B cells [21]. However, PCV2 infection did not significantly affect the expression of *Irf4* and *Cd40*. IL21, which is secreted by activated CD4+ T cells and acts on IL21R on GC B cells, stimulates the production of BCL6 [22]. The expression of BCL6 hinders DNA repair and is beneficial for SHM [23], while our results show a decrease in *Bcl6* expression levels. The expression levels of *Irf4* and *Prdm1*, which promote the differentiation of GC B cells into plasma cells [24,25], did not change significantly in PCV2-induced GC B cells. On the other hand, the expression levels of *Bcl6* and *Pax5*, which inhibit GC B cell differentiation into plasma cells [26], decreased in PCV2-induced GC B cells. This leads to the production of some GC-dependent plasma cells after PCV2 infection. Research has shown that most memory B cells that respond to T cell-dependent antigens arise from GC reactions [13], whereas PCV2 infection results in poor class switch and recall ability in memory B cells. Our results suggest that memory B cells and plasma cells generated by PCV2 stimulation likely perform poorly due to inadequate SHM, affinity maturation, and class switch in the GC response. IgA is found in mucosal tissues such as the respiratory and digestive tracts and is a key component of mucosal immunity. However, PCV2 infection reduces the ability of plasma cells to produce IgA, making PCV2 infectious to relevant target organs and leading to corresponding symptoms such as interstitial pneumonia and diarrhea. Positive selected LZ GC B cells have multiple fates: they can differentiate into recycling GC B cells and return to the DZ for the next round of proliferation and SHM, or they can differentiate into plasma cells or memory B cells and exit the GC response [27,28]. However, PCV2 infection increases MYC expression in LZ GC B cells, promoting their differentiation into recycling GC B cells [29,30], while reducing BACH2 expression hinders their differentiation into memory B cells. The interaction signals between Tfh cells, DCs, macrophages, and LZ GC B cells are weakened, which may be the key reason for the inhibition of LZ GC B cell differentiation caused by PCV2 infection.

Since the symptoms of PCV2 infection in mice resemble those in pigs, including immune suppression, lymphocyte reduction, and interstitial pneumonia, etc., PCV2-infected mouse models are widely used to study PCV2 pathogenesis [31,32,33,34,35], avoiding the obstacle of limited antibody reagents and molecules for identifying porcine B cell subsets [36]. In this study, we confirmed that the serum PCV2 loads increased in mice by time, and the spleen was the major target tissue of PCV2, which is consistent with the previous report that PCV2 also infects the spleen and exhibits lymphoid tissue tropism in mice [33,37]. The histopathologic results showed that PCV2 can induce pathological tissue damage in the spleen of mice, manifested as loss of lymphoid tissue, decreased lymphocytes, and especially sparse arrangement of lymphocytes in lymphoid follicles. This is also consistent with the previous reports [31,32,35]. scRNA-seq technology has such a high cell throughput that 9665 cells for control group mouse and 6477 cells for PCV2 group mouse were captured in this study, respectively. We detected the effect of PCV2 at the cellular level using various experimental methods such as analysis of scRNA-seq and flow cytometry. The effects of PCV2 infection on gene expression at different levels were detected through single-cell RNA sequencing, RT-PCR, Western blot, and flow cytometry. Based on previous findings in pigs, such as PCV2 infection causing a decrease in lymphocytes and leading to immune suppression [4,38], we have clarified the reason for PCV2 infection inhibiting humoral immune responses from the perspective of B cells by studying PCV2-infected mouse models. These results suggest that PCV2 infection may cause humoral immune suppression in pigs via similar mechanisms, providing new insights into PCV2 pathogenesis and vaccine development.

## Data Availability

All raw and processed sequencing data generated of the two mice in this study have been submitted to the NCBI Gene Expression Omnibus (GEO; https://www.ncbi.nlm.nih.gov/geo/; accessed on 20 June 2025) under accession number GSE287478 (https://www.ncbi.nlm.nih.gov/geo/query/acc.cgi?acc=GSE287481, accessed on 20 June 2025).

## Supplementary Materials

The following supporting information can be downloaded at https://www.mdpi.com/article/10.3390/microorganisms13092184/s1, Figure S1: Monitoring of mouse weight; Figure S2: PCV2 infection activates naïve B cells but does not cause their death; Figure S3: PCV2 infection causes germinal center response but does not generate effective humoral immunity; Figure S4: Identification and annotation of Tfh cells; Table S1: Mouse rectal temperature monitoring; Table S2: Real-time PCR primers for mouse genes.

## Abbreviations

The following abbreviations are used in this manuscript:

PCV2	Porcine circovirus 2
GC	germinal center
LZ	light zone
DZ	dark zone
SHM	somatic hypermutation
APC	antigen presenting cell
Tfh	follicular helper T
DCs	dendritic cells
AID	activation-induced cytidine deaminase
scRNA-seq	single-cell RNA sequencing
log2FC	log2(Fold Change)
minute	min
second	s
hour	h
t-SNE	t-distributed stochastic neighbor embedding
NK	natural killer
KEGG	Kyoto Encyclopedia of Genes and Genomes
GO BP	Gene Ontology Biological Process
DEGs	differentially expressed genes
MBC	memory B cell
