# Supplementary material for: PCV2 Infection Represses the Differentiation of Light Zone Germinal Center B Cells by Inhibiting Their Interaction with Helper Cells
Source: Microorganisms. 2025 Sep 18;13(9):2184. doi: 10.3390/microorganisms13092184 (PMC12472866; doi:10.3390/microorganisms13092184)
Supplement: Supplementary file 1 [file microorganisms-13-02184-s001.zip › microorganisms-3839464-supplementary.pdf]

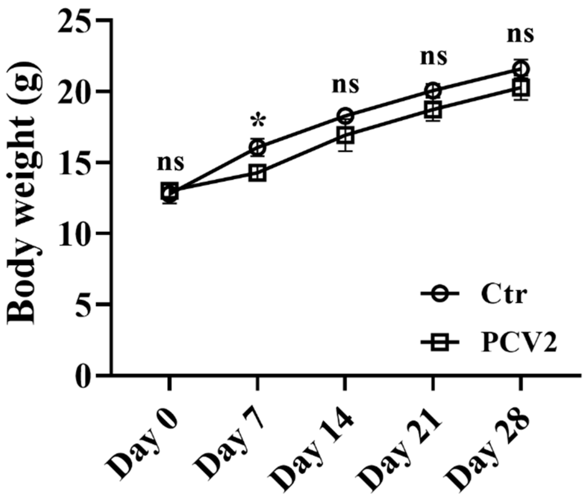


**Figure S1. Monitoring of mouse weight.**

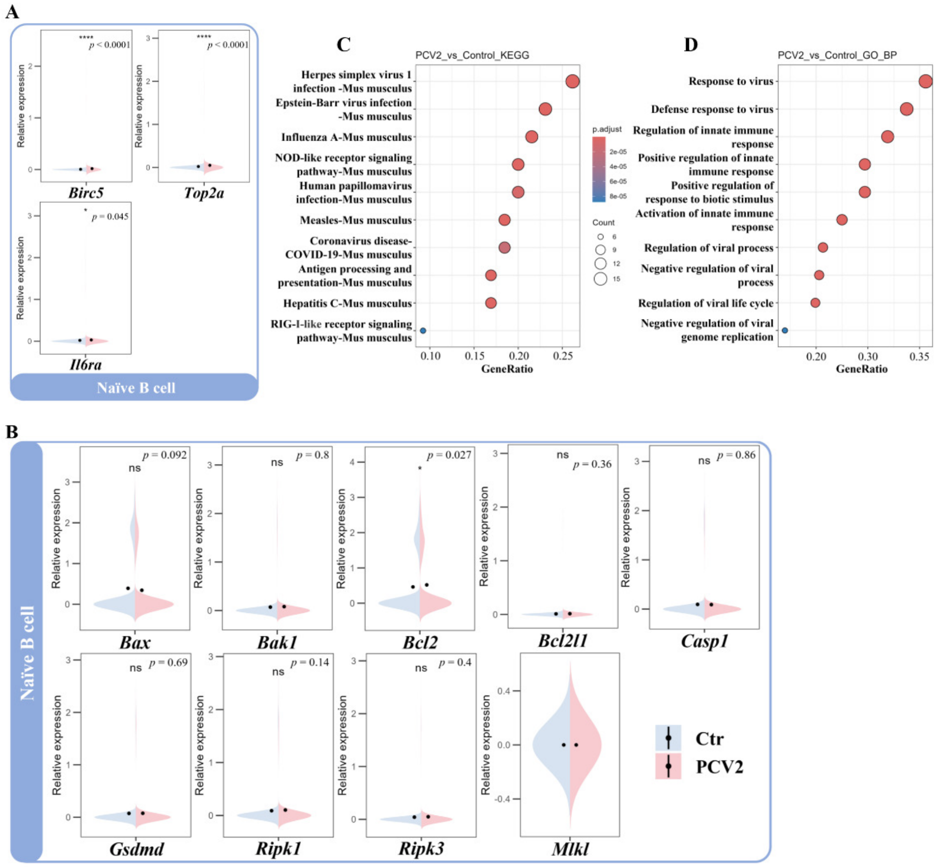


**Figure S2. PCV2 infection activates naïve B cells but does not cause their death.** **(A)** The violin plot shows that the cell replication related genes *Birc5* and *Top2a*, as well as the cell receptor gene *Il6ra*, were highly expressed in PCV2 group vs. the control group in the naïve B cells. **(B)** The violin plot displays the comparative results of the expression of apoptosis positive regulatory genes *Bax5* and *Bak1*, apoptosis negative regulatory genes *Bcl2* and *Bcl2l1*, pyroptosis positive regulatory genes *Casp1* and *Gsdmd*, and necrosis positive regulatory genes *Ripk1*, *Ripk3*, and *Mlkl* in naïve B cells between the control group and the PCV2 group. Kyoto Encyclopedia of Genes and Genomes (KEGG) **(C)** and Gene Ontology Biological Process (GO BP) **(D)** enrichment analysis of the differentially expressed genes (DEGs) of B cells between control group and PCV2 group.

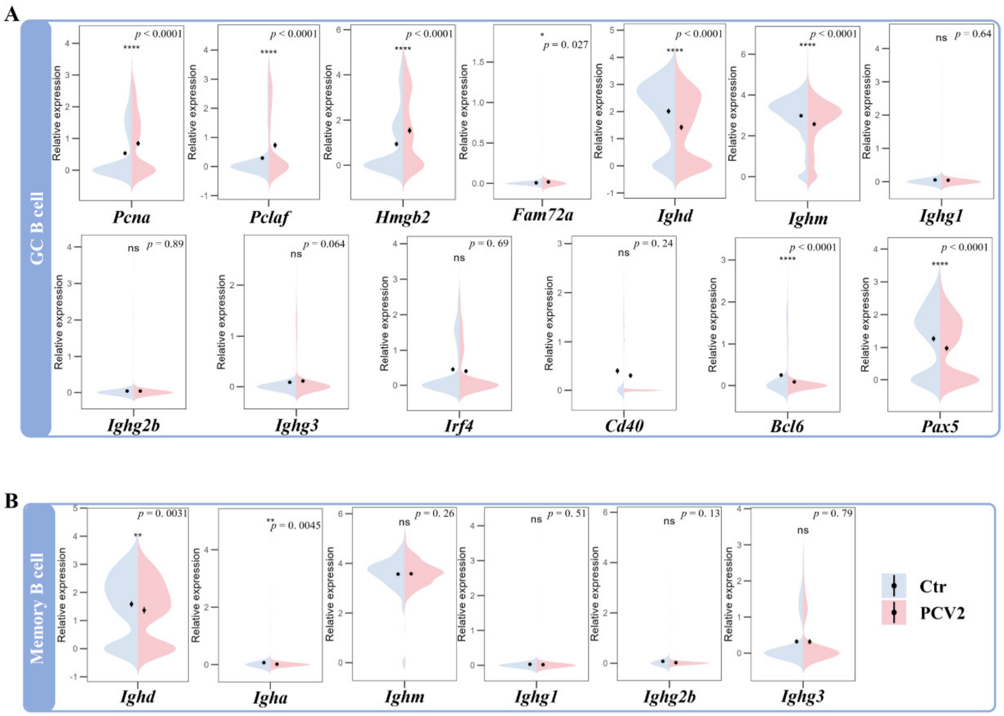


**Figure S3. PCV2 infection causes germinal center response but does not generate effective humoral immunity.**

**(A)** The violin plot displays the comparative results of the expression of cell replication related genes *Pcna*, *Pclaf*, and *Hmgb2*, somatic hypermutation (SHM) related gene *Fam72a*, immunoglobulin related genes *Ighd*, *Ighm*, *Ighg1*, *Ighg2b*, and *Ighg3*, cell response ability related genes *Irf4* and *Cd40*, and *Bcl6* and *Pax5* in GC B cells between the control group and the PCV2 group. **(B)** The violin plot displays the comparative results of the expression of immunoglobulin related genes *Ighd*, *Igha*, *Ighm*, *Ighg1*, *Ighg2b*, and *Ighg3*.

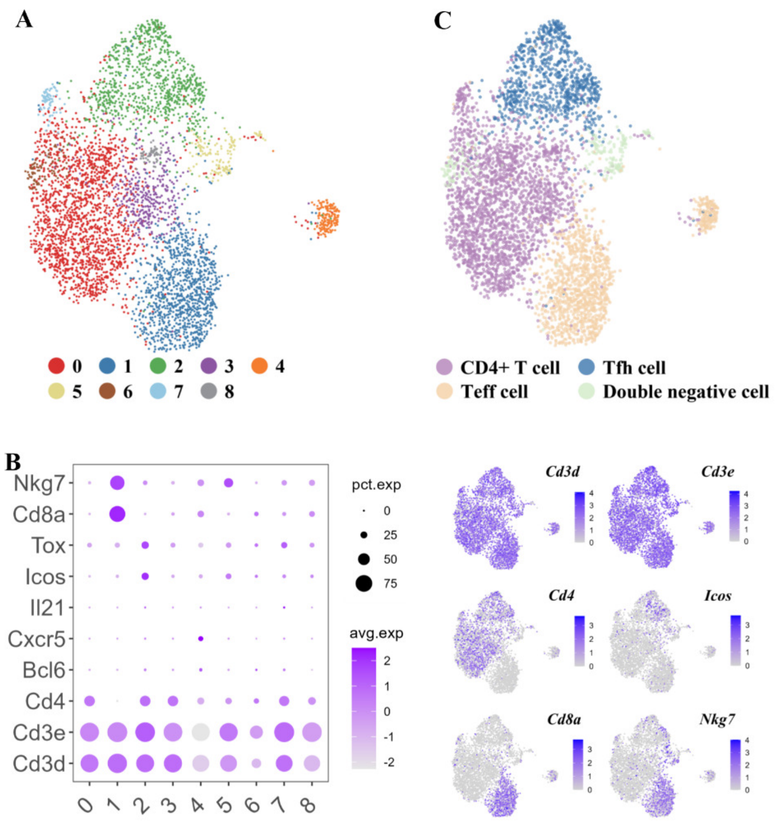


**Figure S4. Identification and annotation of Tfh cells.**

**(A)** UMAP (uniform manifold approximation and projection for dimension reduction) plot visualizing the cluster distribution of the T cells annotated in figure 2C. **(B)** The expression of marker genes *Cd3d* and *Cd3e* for T cells, *Cd4* for CD4+ T cells, *Bcl6*, *Cxcr5*, *Il21*, *Icos*, and *Tox* for Tfh cells, *Cd8a* and *Nkg7* for CD8+ T cells in T cell clusters (left). UMAP projection of some marker genes, including *Cd3d*, *Cd3e*, *Cd4*, *Icos*, *Cd8a*, and *Nkg7* (right). **(C)** UMAP plot showing the annotated CD4+ T cells, Tfh cells, CD8+ T cells, and double negative cells.

**Table S1. Mouse rectal temperature monitoring**

| | Ctr | | | | | PCV2 | | | | |
|---|---|---|---|---|---|---|---|---|---|---|
| **Day 0** | 37.2 | 36.5 | 37.4 | 37.9 | 36.8 | 36.8 | 37.5 | 37.3 | 37.8 | 37.5 |
| **Day 7** | 37.0 | 37.9 | 37.6 | 37.2 | 38.0 | **39.9** | **38.6** | 38.3 | **38.7** | **39.6** |
| **Day 14** | 38.3 | 36.7 | 37.0 | 36.9 | 38.3 | **38.6** | 38.3 | **38.6** | 37.7 | 37.9 |
| **Day 21** | 37.7 | 37.3 | 36.9 | 37.2 | 36.7 | 37.6 | 37.8 | 37.5 | 37.8 | 38.2 |
| **Day 28** | 37.2 | 38.1 | 37.0 | 36.9 | 36.5 | 36.9 | 37.4 | 38 | 36.5 | 37.2 |

**Table S2. Real-time PCR primers for mouse genes (5' to 3')**

| Gene Target | Forward | Reverse |
|---|---|---|
| ***Birc5*** | CCGATGACAACCCGATAGAGG | TGGCTCTCTGTCTGTCCAGT |
| ***Top2a*** | TGGTTTTACGGAGCCAGTTTT | TCACGTCAGAGGTTGAGCAC |
| ***Il6ra*** | TCAACGCCATCTGTGAGTGG | CCCGTTGGTGGTGTTGATTT |
| ***Bax*** | ACTTCACAGGTTGGCATTAGGAAG | ATGGTCACTGTCTGCCATGT |
| ***Bak1*** | CCTTCGGGGTCTTCGTCTTT | ACCGTCACTTGTCACCTGAA |
| ***Bcl2*** | GACTGAGTACCTGAACCGGC | AGTTCCACAAAGGCATCCCAG |
| ***Bcl2l1*** | GCCGGAGATAGATTTGAATAACCT | CCCGGTTGCTCTGAGACATT |
| ***Casp1*** | CTATGGACAAGGCACGGGAC | TCAGCTGATGGAGCTGATTGA |
| ***Gsdmd*** | AGTGCTCCAGAACCAGAACCG | TCTGCCCTGAATGTTCCCATC |
| ***Ripk1*** | GGAAGGCCTCTGAGGACAC | CTTCTCCAGCAGGTCACTGG |
| ***Ripk3*** | GCCTTCCTCTCAGTCCACAC | CTCACCAGAGGAACCGCATA |
| ***Mlkl*** | TCTTTCTGGCAGAGAACGAATCT | TCTTACACCTTCTTGTCCGTGG |
| ***Gapdh*** | ACCCTTAAGAGGGATGCTGC | CCCAATACGGCCAAATCCGT |
